# Regulation of norrin receptor frizzled-4 by Wnt2 in colon-derived cells

**DOI:** 10.1186/1471-2121-8-12

**Published:** 2007-03-26

**Authors:** Kestutis Planutis, Marina Planutiene, Mary Pat Moyer, Anthony V Nguyen, Cherlyn A Pérez, Randall F Holcombe

**Affiliations:** 1 Division of Hematology/Oncology, University of California, Irvine, USA; 2 Incell Corporation, San Antonio, Texas, USA

## Abstract

**Background:**

Norrin is a potent Wnt pathway ligand. Aberrant activation of this signaling pathway can result in colon tumors but the role of norrin-based signaling in the genesis of colon cancer, and its relationship to activation of the pathway by traditional Wnt ligands, is not defined.

**Results:**

Fresh normal human colon tissue and all the cell lines studied expressed mRNA for Fz4, LRP5 and norrin, except Colo205 which lacked Fz4 expression. Canonical Wnt pathway throughput was increased slightly in NCM460 following treatment with Wnt3a CM but was inhibited by Wnt2 and Wnt1. The colon cancer cell line, RKO, responded to Wnt3a CM, Wnt2 and Wnt1 by increasing canonical Wnt pathway throughput. Wnt5a did not affect Wnt pathway throughput in either cell line. Wnt2, but not Wnt3a, abrogated Fz4 expression in NCM460, but not in RKO or another colon cancer cell line, HCT116. This effect on Fz4 was confirmed at both the RNA and protein levels via RT-PCR and a norrin binding assay. The expression of all others 9 Fz receptors did not change after treatment of NCM460 cells with Wnt2.

**Conclusion:**

The data suggests that colonic mucosa and colon tumors may possess two autoregulatory positive Wnt feedback loops, one through canonical signals induced by Wnt:Fz interactions and one through signals resulting from norrin:Fz4 interactions. The latter interactions may be modulated via regulation of Fz4 expression by Wnt2. Retention of Fz4 by cancers, in contrast to the loss of Fz4 by normal mucosal cells, could provide a selective advantage to the tumor cells. Fz4 expression may play a critical role in responses to Wnt signaling in the tumor microenvironment.

## Background

Signaling through the Wnt pathway begins with Wnt ligands, secreted growth factors that interact with a Frizzled (Fz) cell surface transmembrane receptor together with co-receptors of the low-density lipoprotein receptor-related protein (LRP) family to initiate the signal cascade [1-3]. Wnt binding to Fz leads to hyperphosphorylation of Dishevelled [4] which inhibits the activity of glycogen synthase kinase-3 (GSK-3). This, along with destabilization of axin, prevents APC-mediated degradation of β-catenin leading to its accumulation. β-catenin is then available to bind to members of the lymphoid enhancer factor/T-cell factor (LEF/TCF) family of HMG-box transcription factors [5,6] and induce the transcription of growth regulatory genes such as *myc *[7] and *cyclinD1 *[8,9]. Cell surface expression of LRP5/6 appears to be necessary to induce this β-catenin-dependent (canonical) Wnt signaling [10]. In its absence, Wnts may bind to Fz receptors and initiate distinct, non-canonical signaling cascades [11].

Recently, a non-Wnt ligand has been described that binds selectively to Fz receptor subtype-4 (Fz4) and induces canonical Wnt signaling [12-14]. This ligand, norrin, binds with high (nanomolar) affinity to Fz4 and canonical pathway activation via this interaction is dependent on the presence of cell surface LRP5 [12]. Norrin appears to promote angiogenesis. Overexpression in norrin deficient mice induces the growth of ocular capillaries [15] and inactivating mutations results in Norrie disease which is characterized by ocular vascular defects and sensorineural deafness related to impaired cochlear vascularization [16,17]. Abnormalities in Fz4 or LRP5 result in a phenotypically similar condition, familial exudative vitreoretinopathy (FEVR) characterized by defects in retinal vascular development [18-21]. The clinical similarities in vascular phenotypes caused by norrin, Fz4 and LRP5 mutations in humans and mouse models, along with the high specificity of norrin-Fz4 binding support the hypothesis that norrin and Fz4/LRP5 form a functional binding group which is involved in angiogenesis regulation.

Wnt signaling is of paramount importance in the colon, with >85% of sporadic colon cancers associated with activating mutations in APC or β-catenin [22]. Other alterations in Wnt pathway components, including LEF1, Fz receptors and Wnt2 and Wnt5a have been described in colon cancer which may contribute to regulation of Wnt signal throughput [23,24]. Whether norrin or Fz4 have functional relevance in the colon or in the pathogenesis of colon cancer is unknown. In this study, we demonstrate for the first time that the components for norrin:Fz4 signaling are present in the human colon and, in vitro, in normal colonic mucosa-derived cells, endothelial cells and in colon cancer cells. Additionally, in normal colonic mucosa-derived cells, Fz4 expression is regulated by Wnt2. The data suggest that, in vivo, cross-talk between colon cancer, colonic mucosa and, perhaps, colonic mucosa-localized endothelial cells may be an important feature of the tumor microenvironment and may impact tumor-mediated angiogenesis.

## Results

### Expression of norrin and norrin receptor components

**Figure 1:**
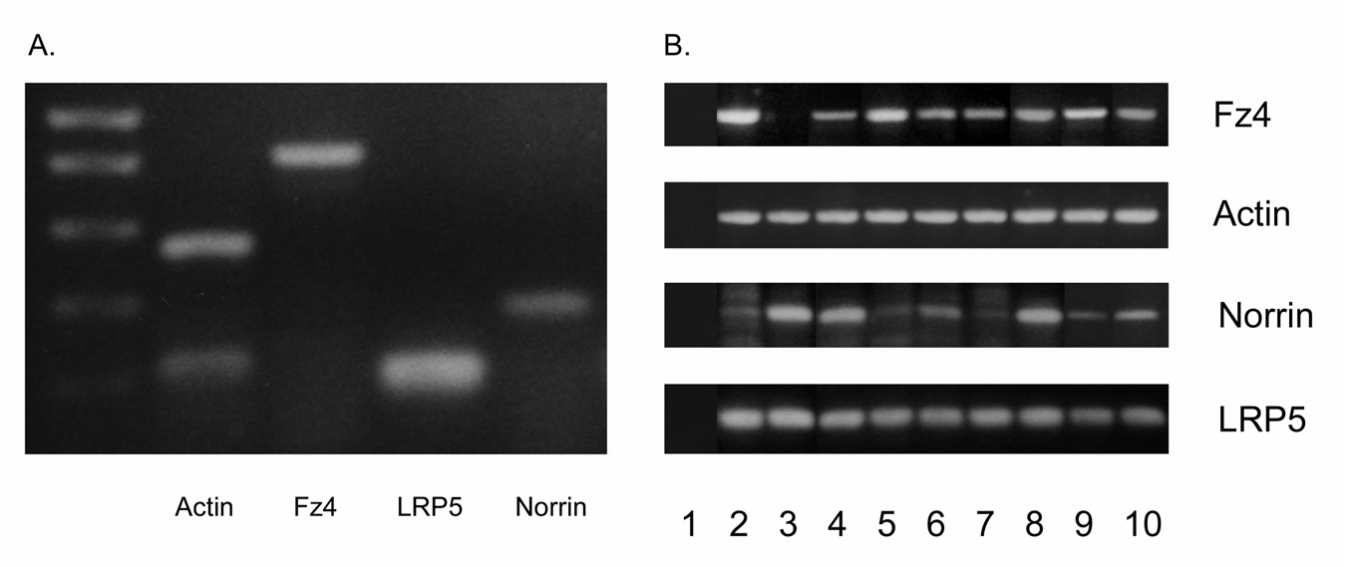
Expression of norrin signaling components. Panel A – RT-PCR for norrin signaling pathway components Fz4 (832 bp), norrin (400 bp), and LRP5 (300 bp) in normal colonic mucosa obtained fresh from colonoscopic biopsy. Molecular weight markers appear in lane 1. β-actin (544 bp) was utilized as a control to confirm RNA quality. Control PCR reactions without reverse transcriptase did not give any PCR products for any RNA sample with any pair of primers (data not shown). Identities of the PCR products were confirmed by sequence analysis. Panel B – Expression defined by RT-PCR of Fz4 (top row), norrin (3^rd ^row) and LRP5 (bottom row) in various cell lines. Lane 1: no template control; lane 2: CSC1; lane 3: Colo205; lane 4: Colo320; lane 5: HCT116; lane 6: HCT116 with additional chromosome 3; lane 7: HT29; lane 8: NCM460 (normal mucosa-derived); lane 9: RKO; lane 10: EaHy926 (endothelial). Fz4, norrin and LRP5 mRNA was detected in all tested cell lines with the exception of Fz4 in Colo205. Actin control is depicted in 2^nd ^row.

Fz4, the norrin receptor, was expressed in normal colonic mucosa, the normal mucosa-derived colon cells, NCM460, an endothelial cell line EaHy926, and each of the tumorigenic colon cancer cell lines, including RKO and HCT116, except Colo205 (Figure 1). LRP5, which is essential for the transmission of norrin-induced canonical Wnt signals, but may not be necessary for noncanonical signaling, was found in fresh colonic tissue and all of the cell lines tested (Figure 1). Similarly norrin mRNA was detected in normal tissue and normal-derived and tumorigenic cell lines. These studies utilized reverse transcriptase (RT)-PCR, not real time PCR, so do not provide quantitative information as to the degree of expression. Confirmation that PCR products represented the specific genes under investigation was attained by direct sequence analysis. Therefore, normal colonic tissue, endothelial cells and normal-derived and cancer-derived colonic cell lines (except for Colo205) express all of the components necessary for norrin-driven canonical (β-catenin-dependent) Wnt signaling.

### Canonical Wnt signaling in NCM460 and RKO

**Figure 2:**
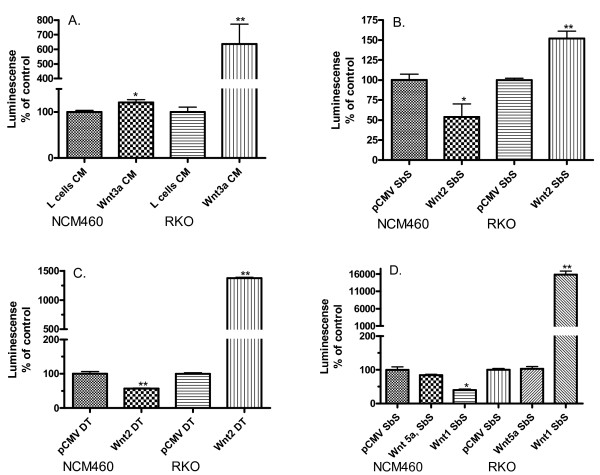
Responsiveness of NCM460 and RKO cells to Wnt stimulation. Wnt pathway throughput is measured following transient transfection of the luciferase-based SuperTopflash reporter construct. All experiments were performed in conjunction with Renilla luciferase determination to control for cell numbers, viability and transfection efficiency. All experiments were repeated at least three times and performed each time in triplicate. Panel A – Incubation with Wnt3a conditioned medium (CM) slightly, but significantly increased Wnt pathway throughput in NCM460 (p = 0.017) and very dramatically in RKO (p < 0.0001). Panel B – Exposure to Wnt2 producing cells in side-by-side (SbS) co-culture decreased Wnt pathway throughput in NCM460 cells (p = 0.027) but increased Wnt pathway throughput in RKO (p < 0.001). Panel C – Direct transfection of a Wnt2 expressin construct decreased Wnt pathway throughput in NCM460 (p < 0.0001) and increased Wnt pathway throughput in RKO (p < 0.0001). Panel D – In SbS co-culture with either Wnt1 or Wnt5a producing cells, Wnt pathway throughput was decreased by Wnt1 (p = 0.002) but unchanged by Wnt5a in NCM460 cells, and increased by Wnt1 (p < 0.0001) but unchanged by Wnt5a in RKO cells.

**Figure 3:**
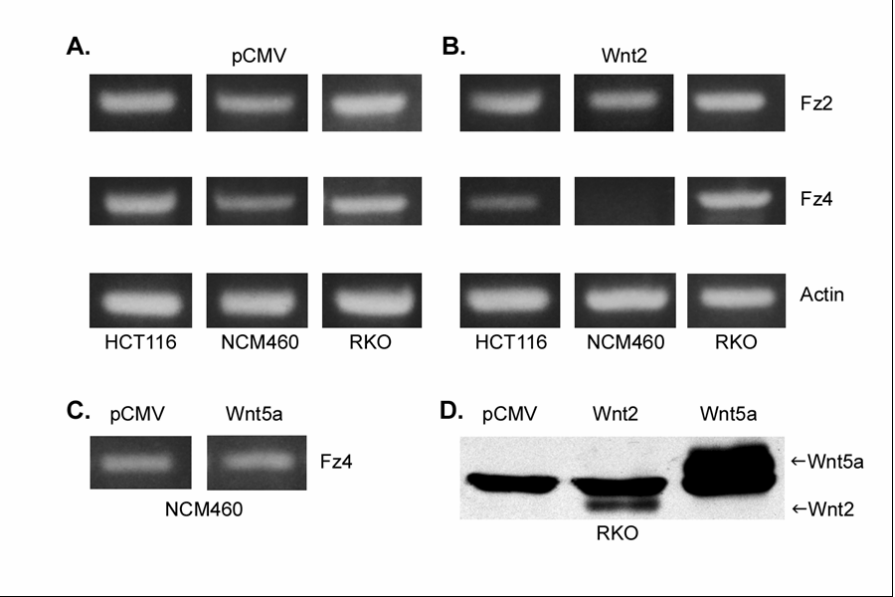
RT-PCR results of Fz4 mRNA expression in normal mucosa-derived NCM460 cells and the colon cancer cell lines HCT116 and RKO following exposure to Wnt-producing cells in co-culture or cells transfected with a non-Wnt-producing pCMV control plasmid. The Fz4 RT-PCR product was 832 bp. All experiments were performed simultaneously with actin primers, producing a 544 bp product for quality control. Fz4 mRNA was downregulated by Wnt2 in normal colonic mucosa-derived NCM460 cells but not in the colon cancer cell lines. No effect on Fz4 expression following exposure to Wnt5a was seen. A. NCM460, HCT116 and RKO cells co-cultured using inserts with sham-transfected cells. B. NCM460, HCT116 and RKO co-cultured with Wnt2-producing cells. C. NCM460 co-cultured with Wnt5a-producing cells. D. Wnt 2 and Wnt5a production by transfected RKO; Western blot. A non-specific band is present in all 3 lanes, including control. The Wnt2 and Wnt5a specific bands are annotated with an arrow.

Several methodologies were utilized to define the activity of canonical Wnt signaling in NCM460 and RKO cells. Incubation with Wnt3a conditioned medium (CM) resulted in a small but statistically significant (p = 0.017) increase in Wnt pathway throughput, measured by luminescence following transient transfection of the Super Topflash reporter construct, in NCM460 cells and a robust and highly significant increase (p < 0.0001) in RKO cells (Figure 2A). NCM460 cells and RKO cells were also incubated in side-by-side co-culture with Cell-tracker-labeled RKO cells which had been previously transfected with a Wnt2 expression plasmid (RKO/Wnt2). Wnt2 production by these cells in culture was confirmed by Western blot of RKO/Wnt2 cell lysates (see Figure 3D). Prior studies demonstrated that Wnt 2 induced nuclear β-catenin translocation, a marker of canonical Wnt pathway activation in RKO cells (data not shown). RKO cells responded with a highly significant increase (p < 0.001) in Wnt throughput but NCM460 cells exhibited decreased Wnt activity (p = 0.027; Figure 2B). To confirm this effect of differential response to Wnt2 between the two cell lines, with a different methodology, NCM460 cells and RKO cells were directly transfected with a Wnt2 expression construct. These experiments confirmed a significant reduction (p < 0.0001) in Wnt throughput in NCM460 and increase in RKO cells (Figure 2C). Canonical Wnt pathway throughput in these cells was further examined in side-by-side co-culture with Wnt5a and Wnt1 producing cells. Exposure to Wnt5a, which is known to signal via non-canonical pathways in several experimental systems [27], did not affect canonical Wnt activity in either cell. Exposure to Wnt1, a strong inducer of canonical Wnt signals [28], significantly increased Wnt throughput in RKO cells (p < 0.0001) but, similar to Wnt2, decreased Wnt throughput in NCM460 cells (p = 0.002, Figure 2D).

### Effect of Wnt2 on Fz4 expression

**Figure 4:**
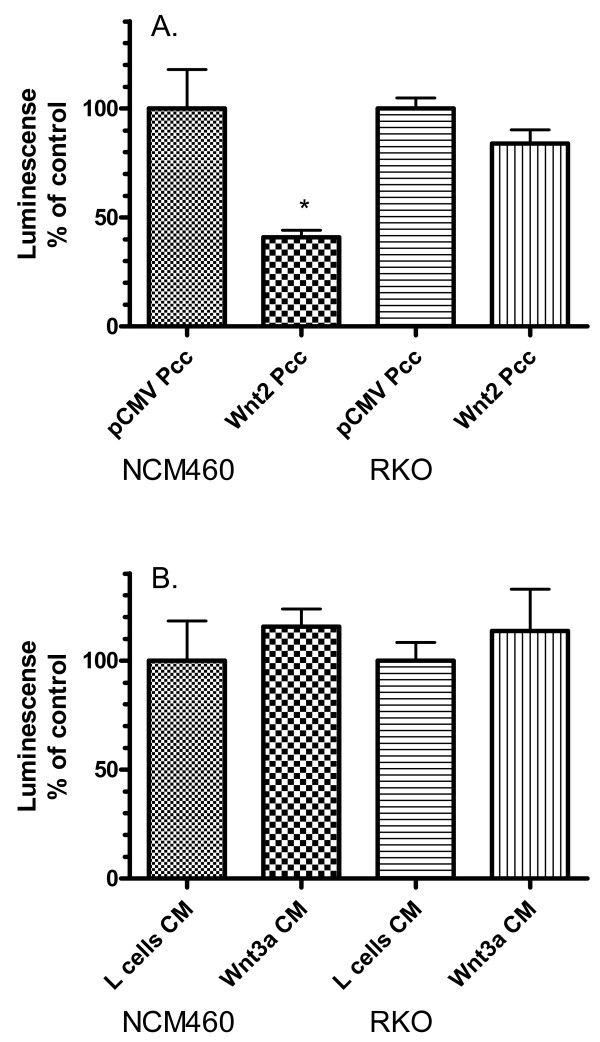
Norrin binding activity of NCM460 and RKO cells after partitioned co-culture. Norrin binding was downregulated by Wnt2, but not Wnt3a in normal colonic mucosa-derived NCM460 cells. Each experiment was repeated three times with six (panel A) or five (panel B) replicates for each data point. A. Comparison of norrin binding in NCM460 (left) and RKO (right) after exposure to sham-transfected cells and Wnt2-producing cells in partitioned co-culture (Pcc). The decrease in norrin binding in NCM460 is highly statistically significant (p < 0.01). B. Comparison of norrin binding in NCM460 (left) and RKO (right) after exposure to control (L-cell) and Wnt3a conditioned medium (CM). No statistically significant difference was seen.

NCM460, HCT116 and RKO cells were incubated in partitioned co-culture with Wnt2 producing cells. Under these conditions, Wnt2 eliminates Fz4 mRNA expression in NCM460 cells but not in RKO cells or HCT116 cells (Figure 3A, 3B). The presence of Fz4 mRNA was determined by RT-PCR with specific oligonucleotide primers. The loss of detectable Fz4 mRNA in NCM460 was uniformly confirmed in six separate experiments and was seen only when co-cultured with Wnt2-producing cells and not when co-cultured with sham-transfected (pCMV plasmid containing) cells. The effect was not seen following co-culture with cells transfected with a Wnt5a expression construct (Figure 3C). Expression of Fz1, 2, 3, 5, 6, 7, 8, 9 and 10 was not affected by Wnt 2 (Fz2 represented in Figure 3B). To determine whether the loss of Fz4 message resulted in changes in Fz4 protein expression at the cell surface, a norrin-binding assay, utilizing soluble AP-3myc norrin, was performed (Figure 4A). Exposure of NCM460 to Wnt2 significantly reduced norrin binding activity (p < 0.01), indicating a reduction in the cell surface expression of Fz receptor capable of binding norrin and suggesting that this is directly resultant from the abrogation of Fz4 gene expression. No significant change in norrin binding activity was seen for RKO cells after exposure to Wnt2, consistent with the RT-PCR data. Norrin binding activity in NCM460 and RKO was not affected by incubation with Wnt3a CM (Figure 4B).

## Discussion and conclusion

Study of the extracellular and cell surface components of the Wnt pathway in tumors and adjacent tissues has received increased attention with the recognition that, even in the face of activating mutations in the pathway present in many cancers, the Wnt signal throughput can be modified by promoting or inhibiting signaling through the cell surface receptors [29,30]. In this report, we demonstrate that the components for an alternative mechanism of Wnt pathway activation, via norrin and Fz4, are fully present in normal colonic mucosa, a normal-mucosa derived cell line, an endothelial cell line and most colon cancer cell lines. In the tumor microenvironment, tumor-derived or mucosa-derived norrin may be available for interaction with mucosal and endothelial Fz4. The availability of LRP5 implies that signaling through norrin:Fz4 interactions could lead to canonical pathway activation [12].

This is the first report in a mammalian system where a specific Wnt ligand (Wnt2) decreases the expression of a specific Fz receptor subtype (Fz4), without affecting the expression of other Fz receptors. The effect was confirmed at both the RNA level, by reverse transcriptase PCR, and at the protein level, with a norrin binding assay. Furthermore, we demonstrate a degree of tissue specificity: Wnt2 decreases Fz4 expression in the normal-derived cells but not in β-catenin-mutated (HCT116) or non-APC and non-β-catenin mutated (RKO) colon cancer cells. Since norrin activates canonical Wnt signaling [13,14], the retention of Fz4 expression by the colon cancer-derived cell lines would favor continued stimulation with this ligand, even in the presence of Wnt2 which is known to be overexpressed by many colon cancers [24]. On the other hand, downregulation of Fz4 in normal mucosa may protect it from stimulation by norrin or other Wnt ligands present within the tumor microenvironment. Wnt2 did not downregulate the expression of other frizzled receptors, including Fz2. Therefore, both normal and tumorigenic cells would retain the capacity to respond to stimulation by extracellular Wnt ligands utilizing Fz2 or other Fz receptors.

Autoregulation within the Wnt pathway, whereby Wnt signaling affects the expression of genes encoding proteins within its own pathway has been noted previously. Wnt3a has been shown by microarray analysis in human teratocarcinoma cells to increase the expression of Fz7 [31] and Wg (wingless) is known to inhibit the expression of DFz2 in *Drosophila *[32]. This is the first report, however, describing Wnt-dependent regulation of the norrin receptor, Fz4, suggesting the existence of crosstalk between the Wnt- and norrin-signaling pathways. The lack of effect of Wnt3a (measured by norrin binding assay) and Wnt5a (measured by RT-PCR) on Fz4 expression suggests that the downregulation is Wnt2 specific. Mikels and Nusse [33] have shown that Wnt5a can signal through canonical or non-canonical pathways, dependent on the cellular context, with non-canonical signals antagonist to canonical signaling. Wnt2 has been previously described to signal both canonically [34] and non-canonically [35]. Our data supports the idea that the intracellular response to Wnt2 may also be context dependent, in that this ligand reduced Wnt throughput, and abrogated Fz4 expression, in NCM460 but increased Wnt throughput, and did not abrogate Fz4 expression, in RKO and HCT116. Whether this difference relates to the origin of the cells (normal mucosa vs. tumor), or whether the decrease in Fz4 expression is restricted to cells like NCM460 which exhibit a relatively poor response to canonical signals, remains to be defined. Interestingly, stimulation with a strongly canonical Wnt ligand, Wnt1 [28], also led to a reduction in Wnt signal throughput in NCM460, suggesting that signaling induced by this ligand can also be context dependent.

The precise role of norrin-based signaling in gastrointestinal tract and in the tumor microenvironment is unknown. Our data on cell lines in vitro suggests that colonic tumors may have available two autoregulatory stimulatory loops, one which operates via interactions between Wnt2 (or other Wnt ligands) and various Fz receptors and one that operates via interactions between norrin and Fz4. While human cancers have been shown to overexpress various Wnt ligands [24], the level of norrin expression in human colon cancers remains to be defined.

Regulation of Fz4 expression in normal colonic mucosa may be critical for modulation of Fz4-based (both Wnt and norrin initiated) signaling in the tumor microenvironment. Inhibition of Fz4 expression due to tumor-derived Wnt2 would decrease Fz4-dependent signal throughput, whereas tumor- or normal colonic mucosa-derived norrin would promote Fz4-dependent signal throughput. A balance between these opposing forces may be important for maintaining colonic mucosal integrity. Because of the known effects of Wnt signaling on gastrointestinal stem cells [1,36], we would also postulate that control of Fz4 expression on colonic mucosal stem cells may play some role in regulating stem cell differentiation.

Additional studies are underway to define whether Wnt2 has similar effect on endothelial cell Fz4 in tumor vasculature as it does on normal colon mucosa-derived cells. Given the known relationship of mutated norrin and Fz4 to abnormal vascularization in the eye and ear [13,14], perturbations in norrin:Fz4 signaling may be one explanation for the disorganized and "leaky" neovascularization which accompanies tumor-stimulated angiogenesis [37]. Our data in an endothelial cell line indicate that all of the components necessary for norrin:Fz4 signaling are present. Finally, the role of norrin in the colon cancer needs further exploration in order to define whether this arm of the "Wnt" signaling pathway may be a suitable target for cancer prevention or cancer therapy.

## Methods

### Human tissue acquisition

Biopsy specimens of normal colonic tissue were obtained during diagnostic colonoscopy following informed consent under an IRB-approved protocol at the H.H. Chao Comprehensive Digestive Disease Center at the University of California, Irvine. Tissue was incubated at +4°C in RNAlater (Ambion, Austin, Texas) overnight then stored frozen at -80°C. Tissue was subsequently homogenized and mRNA was isolated with a TRIzol reagent (Invitrogen, Carlsbad, California) according to standard protocols.

### Normal colonic mucosa-derived and colon cancer cell lines

RKO, a human colon cancer cell line, was kindly provided by Dr. Parker (UCLA). It was maintained in culture in RPMI1640 media at 5% CO_2 _with 10% fetal bovine serum (FBS). CSC1 and the normal mucosa-derived colon cell line, NCM460 were obtained from Incell Corporation (San Antonio, Texas). These cell lines were maintained in M3 Base cell culture medium complete (Incell, catalog number M300A-500) with 10% FBS. All other cell lines were obtained from the American Type Culture Collection (ATCC, Manassas, Virginia).

### Side-by-side co-culture

RKO cells were plated in six-well dishes, grown until they are ~60% confluent and transfected with either Wnt2 or Wnt5a expression constructs (Upstate Biotechnology, Lake Placid, New York) or, as a control, pCMV vector without insert (Stratagene, La Jolla, California). For transfection, 2 μg per well of DNA was added in 100 μl of Opti-MEM I Reduced Serum Medium (Invitrogen, catalog No. 11058-021). Separately 6 μl of Lipofectamine 2000 (Invitrogen, catalog No. 11668-019) were added to 100 μl of Opti-MEM I. These two preparations were combined, and mixed on shaker at room temperature for 20 minutes. The cells were covered with 0.8 ml DMEM with 10% fetal bovine serum. After the addition of the DNA-Lipofectamine mixture, the cells were incubated overnight. After rinsing, the cells were placed in culture with either Super Topflash or Ranilla luciferase transfected RKO cells or NCM460 cells.

### Partitioned co-culture

For analysis of the effect of Wnt ligands on Fz4 expression and for the norrin binding assay, a partitioned co-culture system was utilized. RKO cells were transfected as indicated above, and layered in 6 well plates. Untransfected NCM460, HCT116 or RKO cells were plated on 0.4 μm pore size PET track-etched membrane (Becton Dickinson Biosciences, San Jose, California, catalog No. 35-3090), and grown until 30% confluence. These membrane-bottomed receptacles served as cell culture inserts. The inserts with target cells were placed over transfected Wnt-producing or sham-transfected RKO cells, and co-cultured for 24 hours. In this system, proteins secreted by the transfected cells can directly influence target cells because they share the same media. In the partitioned co-culture system, Wnt2 and Wnt5a protein are secreted by the transfected cells (prepared as described above). By utilizing the HA-tag and specific antibodies, they can be detected by Western blotting. Thus, this system permits the measurement of cellular effects in the target cells of secreted Wnt ligands and maintains a separate population of untransfected cells which can be harvested for analysis. Following co-culture, target cells were harvested from the membranes for analysis of Fz4 expression and for the norrin binding assay.

### Conditioned Medium

Wnt3a conditioned medium (CM) was prepared according to procedures perfected in the laboratory of Dr. R Nusse [25,26].

### Reverse transcriptase PCR

RNA was purified using TRIzol reagent according to the manufacturer's procedure (Invitrogen) and dissolved in 20 μl of DEPC-treated water. 1 μg of the RNA was treated with Deoxyribonuclease I (Invitrogen, catalog No. 18068-015). Reverse transcriptase PCR was done applying SuperScript One-Step RT-PCR for Long Templates (Invitrogen, catalog No. 11922-028) with PTC programmable thermal controller (MJ Research, Inc.) according to the manufacturers' procedures. Primer sets for actin (control for RNA sample's quality), Fz4, norrin and LRP5 were kindly provided by Drs. Tim Byun, Xiaolin Zi and Yi Guo. The thermo-cycler parameters were: 30 min, 55°C; 2 min 94°C; 40 cycles of: 15 sec 94°C, 30 sec 55°C and 1 min 72°C; final extension of 10 min, 72°C. Reaction products were analyzed by electrophoresis on 0.8% agarose gels.

### Quantitative estimation of luciferase activity

To evaluate Wnt pathway throughput following transient transfection of a Super Topflash reporter construct which contains repeated LEF/TCF binding sites preceding a luciferase reporter, a Dual-Glo Luciferase Assay System (Promega, Madison, WI) was used, following manufacturers instructions and utilizing 50 μl of firefly luciferase substrate. After incubation at room temperature for 10 min, the luminescence was measured using ML 3000 Microtiter Plate Luminometer (Dynatech Laboratories, Orlando, FL). After that 50 μl of Renilla luciferase substrate were added, and the luminescence was measured again. (Renilla luciferase expression construct pRstF was kindly provided by Dr. R. Gesteland from University of Utah.) Renilla luciferase data were used to normalize for transfection efficiency and changes in cell survival and growth.

### Norrin binding assay

After 24 hours of partitioned co-culture, cells in the inserts were transferred to new 6 well plates on ice. Norrin-conditioned medium, generated after 24 hours of culture in cells transfected with an alkaline phosphatase-3myc-mNorrin conjugate expression construct, generously provided by Dr. J. Nathans, was added both inside (1 ml) and outside (2 ml) of the insert. The cells were kept on ice for 1 hour, rinsed, and treated with 0.25 ml 1% Triton X-100 in HBS. A disposable cell scraper was utilized to collect the cells. A Great EscApe SEAP kit (Becton Dickinson, catalog No. 631701) was used to measure the alkaline phosphatase bound to the target cells. 15 μl of the lysate was added to 45 μl 1× Dilution Buffer in 96 well black assay plate with clear bottom, tissue culture treated (Corning Incorporated, New York, catalog No. 3603). The plate was wrapped into a plastic wrap and incubated for 30 min 65°C to inactivate endogenous cellular phosphatases. After the addition of the substrate, chemiluminescence was measured using an ML 3000 Microtiter Plate Luminometer (Dynatech Laboratories, Chantilly, Virginia). This norrin-alkaline phosphatase chimera does not induce Wnt signaling and is utilized only to define the degree of Fz4 expression via a binding assay.

### Western blot analysis

Whole cell protein extracts from transfected cells were prepared using a 1× loading buffer solution containing 5 mM Tris-Cl, pH 6.8, 2% SDS and 10% glycerol and separatated by SDS-polyacrylamide gel electrophoresis. Following transfer to nitrocellulose, blots were treated with 5% dried mild in 1× PBS to block non-specific binding, washed, and incubated with primary antibody in 0.1% Tween-20/PBS for 1 hour at room temperature and subsequently overnight at 4°C. Transfection efficiency analysis was performed in the transfected RKO cells using an anti-HA polyclonal primary antibody (Santa Cruz Biotechnology) at a dilution of 1:1000. All membranes were re-blotted with an anti-actin polyclonal antibody at a dilution of 1:1000 to allow for normalization between lanes and experiments. After washes, blots were incubated with a conjugate of horseradish-peroxidase secondary antibodies in 1× PBS/0.1% Tween-20. Peroxidase localization was determined using a chemiluminescence kit (Pierce, Woburn, MA). Intensity of bands was defined by light transmittance densitometry (BioRad, Hercules, CA).

### Statistical considerations

Means and standard deviations for all experimental data were calculated. Comparisons between different experimental conditions were made using a 2-tailed t-test for unpaired samples with an α < 0.05 for significance.

## Authors' contributions

KP and MP carried out partitioned co-culture and side-by-side co-cultures experiments, norrin binding assays, and all luciferase assays. KP additionally supervised activities of AVN and CAP. MPM developed and provided the NCM460 cell line. AVN assisted with tissue acquisition and qRT-PCR experiments. CAP participated in evaluation of expression of norrin in human-derived tissues and cell lines. RFH provided oversight of all of the research and participated in its design and coordination. All authors read and approved the final manuscript.
